# A 3-year natural history of orthostatic blood pressure dysregulation in early Parkinson’s disease

**DOI:** 10.1038/s41531-023-00546-5

**Published:** 2023-06-21

**Authors:** Sang-Won Yoo, Yoon-Sang Oh, Dong-Woo Ryu, Seunggyun Ha, Yuna Kim, Ji-Yeon Yoo, Joong-Seok Kim

**Affiliations:** 1 Department of Neurology, College of Medicine, The Catholic University of Korea, Seoul, Republic of Korea; 2 Division of Nuclear Medicine, Department of Radiology, College of Medicine, The Catholic University of Korea, Seoul, Republic of Korea

**Keywords:** Parkinson's disease, Neurological manifestations

## Abstract

In Parkinson’s disease (PD), cardiovascular dysautonomia accumulates with disease progression, but studies are lacking on the natural history behind each subtype except orthostatic hypotension. This study investigated the early natural history of orthostatic blood pressure (BP) subtypes in PD. Two hundred sixty-seven early PD patients were included. Their cardiovascular functions were assessed by head-up tilt-test and ^123^I-metaiodobenzylguanidine scintigraphy. All patients were classified as having supine hypertension (SH), orthostatic hypertension (OHT), delayed orthostatic hypotension (dOH), or orthostatic hypotension (OH) according to consensus criteria. The patients were assigned to one of three groups: extreme BP dysregulation (BP_extreme_), mild BP dysregulation (BP_mild_), and no BP dysregulation (BP_none_) according to their orthostatic BP subtypes. The autonomic functions of 237 patients were re-assessed after approximately 3 years. Among initially enrolled subjects, 61.8% of the patients showed orthostatic BP dysregulation: 29.6% in the BP_extreme_ group and 32.2% in the BP_mild_ group. At follow-up, the BP_extreme_ group increased in number, while the BP_mild_ group diminished. Two-thirds of the initial BP_extreme_ patients maintained their initial subtype at follow-up. In comparison, 40.7% of the initial BP_mild_ patients progressed to the BP_extreme_ group, and 32.4% and 14.7% of the initial BP_none_ group progressed to BP_extreme_ and BP_mild_ groups, respectively. Cardiac denervation was most severe in the BP_extreme_ group, and a linear gradient of impairment was observed across the subtypes. In conclusion, various forms of positional BP dysregulation were observed during the early disease stage. SH and OH increased with disease progression, while OHT and dOH decreased, converting primarily to SH and/or OH.

## Introduction

Parkinson’s disease (PD) is a neurocardiologic disorder that includes a spectrum of positional blood pressure (BP) dysregulations ranging from hypertensive to hypotensive states^1^. BP dysregulation is a continuum in its respective body position, demarcated by operational criteria. These positional BP fluctuations can take the form of supine hypertension (SH), orthostatic hypertension (OHT), delayed orthostatic hypotension (dOH), or orthostatic hypotension (OH)^2–5^.

The pathophysiology of orthostatic BP dysregulation is multifaceted and involves central and peripheral catecholaminergic deficits^6,7^, and pathobiological inter-relay between dOH and OH has been suggested^3,8^. Delayed OH is a mild form of OH^5^. These findings suggest that the range of positional hemodynamics represents different levels of adrenergic derangement. Also, each orthostatic subtype can influence the clinical outcomes negatively^9–12^, highlighting the need to investigate the characteristics of each.

Many studies have focused on the neurobiology and outcome of OH in PD^6^; dOH, SH, and OHT have received relatively little attention until recently^3,5^, and their inter-relationships have been ignored. This research aimed to describe the features of orthostatic subtypes in early de novo PD. We focused on the interrelationships of these subtypes through disease progression and on their early changes to minimize confounders of comorbidities and dopaminergic medication use.

## Results

### Clinical and autonomic characteristics

The clinical and autonomic features of the patients are summarized in Tables 1 and 2. Seventy-nine of 267 patients (29.6%) were classified as BP_extreme_, and 86 (32.2%) were classified as BP_mild_ at enrollment. Overall, 61.8% of the total population had orthostatic BP dysregulation. The mean age at diagnosis was 66.9 ± 9.1 years, and 128 (47.9%) were female. Disease duration at diagnosis was 13.3 ± 10.8 months, and patients were monitored clinically through an average period of 61.4 ± 21.8 months (Supplementary Fig. 1). The age at diagnosis was oldest in the BP_extreme_ group, and this tendency persisted during follow-up. The total follow-up period and levodopa equivalent daily dose (LEDD) did not differ across subgroups, and there were no differences among groups in the types of drugs used to treat PD symptoms at each patient’s endpoint. Antihypertensive classes were not different, with fewer than six participants in each subgroup taking beta-blockers.

**Table 1:** Participant baseline clinical demographics.

	Initial						Follow-up^+^					
	PD	BP_extreme_^a^	BP_mild_^b^	BP_none_^c^	*P*-value	Posthoc^d^	PD	BP_extreme_^a^	BP_mild_^b^	BP_none_^c^	*P*-value	Posthoc^d^
	*n* = 267	*n* = 79	*n* = 86	*n* = 102			*n* = 237	*n* = 121	*n* = 38	*n* = 78		
Age at diagnosis, years	66.9 ± 9.1	70.1 ± 8.0	64.8 ± 9.2	66.3 ± 9.1	< 0.001	a > b***, a > c*	66.7 ± 9.1	68.5 ± 7.6	64.2 ± 11.0	65.0 ± 9.8	0.008	a > b*
Sex, female, *n* (%)	128 (47.9)	33 (41.8)	44 (51.2)	51 (50.0)	0.419		116 (48.9)	48 (39.7)	20 (52.6)	48 (61.5)	0.010	
Body mass index, kg/m^2^	24.1 ± 3.0	23.6 ± 2.8	24.2 ± 2.8	24.3 ± 3.3	0.215		24.0 ± 3.0	23.7 ± 3.0	24.8 ± 3.2	24.1 ± 2.9	0.123	
Disease duration at diagnosis, months	13.3 ± 10.8	13.2 ± 10.5	13.6 ± 11.8	13.1 ± 10.3	0.939		13.0 ± 10.8	13.9 ± 11.6	15.2 ± 10.6	10.7 ± 9.2	0.052	
Total follow-up period, months	61.4 ± 21.8	61.2 ± 21.9	63.2 ± 22.0	59.9 ± 21.6	0.581		62.0 ± 21.5	62.3 ± 20.9	58.3 ± 23.4	63.2 ± 21.7	0.493	
Hypertension, *n* (%)	109 (40.8)	32 (40.5)	38 (44.2)	39 (38.2)	0.716		99 (41.8)	55 (45.5)	12 (31.6)	32 (41.0)	0.311	
Calcium channel blocker, *n* (%)	54 (49.5)	17 (54.8)	16 (42.1)	21 (53.8)	0.483		—	—	—	—	—	
Angiotensin II receptor blocker, *n* (%)	76 (69.7)	21 (65.6)	26 (68.4)	29 (74.4)	0.777		—	—	—	—	—	
Beta blocker, *n* (%)	14 (12.8)	5 (15.6)	5 (13.2)	4 (10.3)	0.674		—	—	—	—	—	
Diuretics, *n* (%)	23 (21.1)	9 (28.1)	7 (18.4)	7 (17.9)	0.486		—	—	—	—	—	
Diabetes mellitus, *n* (%)	36 (13.5)	15 (19.0)	5 (5.8)	16 (15.7)	0.026		30 (12.7)	21 (17.4)	3 (7.9)	6 (7.7)	0.109	
Dyslipidemia, *n* (%)	75 (28.1)	22 (27.8)	22 (25.6)	31 (30.4)	0.771		69 (29.1)	39 (32.2)	11 (28.9)	19 (24.4)	0.504	
Non-smoker, *n* (%)	256 (95.9)	77 (97.5)	80 (93.0)	99 (97.1)	0.365		227 (95.8)	114 (94.2)	36 (94.7)	77 (98.7)	0.246	
Chronic kidney disease, *n* (%)	1 (0.4)	0 (0.0)	1 (1.2)	0 (0.0)	0.618		—	—	—	—		
Coronary artery disease, *n* (%)	19 (7.1)	6 (7.6)	5 (5.8)	8 (7.8)	0.871		—	—	—	—		
Converted MDS-UPDRS Part II	6.6 ± 4.3	7.2 ± 4.7	6.1 ± 4.6	6.6 ± 3.6	0.247		8.2 ± 5.1	8.7 ± 5.3	7.3 ± 4.7	7.8 ± 5.1	0.263	
Converted MDS-UPDRS Part III	18.5 ± 9.1	19.1 ± 9.4	18.5 ± 10.4	18.0 ± 7.8	0.735		19.5 ± 11.0	21.1 ± 12.2	18.6 ± 12.0	17.5 ± 7.6	0.044	a > c*
H&Y stage, median (IQR)	2 (1.0)	2 (0.0)	2 (1.0)	2 (1.0)	0.021	a < b*	2 (0.0)	2 (0.0)	2 (1.0)	2 (1.0)	0.276	
Motor phenotype´					0.007						0.099	
TD´, *n* (%)	83 (31.1)	13 (16.5)	37 (43.0)	33 (32.4)			38 (16.0)	15 (12.4)	8 (21.1)	15 (19.2)		
PIGD, *n* (%)	157 (58.8)	55 (69.6)	42 (48.8)	60 (58.8)			179 (75.5)	99 (81.8)	28 (73.7)	52 (66.7)		
Intermediate, *n* (%)	27 (10.1)	11 (13.9)	7 (8.1)	9 (8.8)			20 (8.4)	7 (5.8)	2 (5.3)	11 (14.1)		
Motor phenotype˝					0.293						0.111	
TD˝, *n* (%)	27 (10.1)	4 (5.1)	13 (15.1)	10 (9.8)			6 (2.5)	1 (0.8)	1 (2.6)	4 (5.1)		
AR, *n* (%)	127 (47.6)	38 (48.1)	38 (44.2)	51 (50.0)			161 (67.9)	87 (71.9)	21 (55.3)	53 (67.9)		
Mixed, *n* (%)	113 (42.3)	37 (46.8)	35 (40.7)	41 (40.2)			70 (29.5)	33 (27.3)	16 (42.1)	21 (26.9)		
Total LEDD, mg	—	—	—	—	—		449.9 ± 218.3	451.2 ± 192.7	469.8 ± 206.7	438.2 ± 259.5	0.780	
Levodopa/decarboxylase inhibitor, *n* (%)	—	—	—	—	—		224 (94.5)	115 (95.0)	37 (97.4)	72 (92.3)	0.585	
Dopamine agonist, *n* (%)	—	—	—	—	—		198 (83.5)	100 (82.6)	32 (84.2)	66 (84.6)	0.971	
MAO-B inhibitor, *n* (%)	—	—	—	—	—		37 (15.6)	19 (15.7)	6 (15.8)	12 (15.4)	1.000	
Amantadine, *n* (%)	—	—	—	—	—		6 (2.5)	4 (3.3)	0 (0.0)	2 (2.6)	0.858	
COMT inhibitor, *n* (%)	—	—	—	—	—		1 (0.4)	1 (0.8)	0 (0.0)	0 (0.0)	1.000	

Data are shown as mean ± standard deviation unless remarked otherwise. One-way analysis of variance (ANOVA) or Kruskal-Wallis tests were performed for continuous or ordinal variables and Fisher’s exact test was used for categorical variables to compare between-group differences. Drug classes for the treatment of hypertension or Parkinson’s disease were used as single drugs or in combination depending on each patient. The percentage was calculated as the number of a particular drug class user divided by the total patients in that category. *BP*_*extreme*_ extreme BP dysregulation group, *BP*_*mild*_ mild BP dysregulation group, *BP*_*none*_ no BP dysregulation group, *MDS-UPDRS* Movement Disorder Society-Unified Parkinson′s Disease Rating Scale, *H&Y* Hoehn and Yahr, *TD*′ tremor dominant, *PIGD* postural instability/gait difficulty, *AR* akinetic-rigid, *LEDD* levodopa equivalent daily dose, *MAO-B* Monoamine oxidase-B, *COMT* Catechol-O-methyltransferase, *bpm* beat-per-minute, *IQR* interquartile range.

^d^Multiple comparisons were adjusted by Tukey, Games-Howell, or Dwass-Steel-Critchlow-Fligner methods, as appropriate.

+ Follow-up loss (*n* = 30) is not shown in the table.

*p < 0.05; **p < 0.01; ***p < 0.001.

**Table 2:** Participant autonomic characteristics.

	Initial						Follow-up^+^					
	PD	BP_extreme_^a^	BP_mild_^b^	BP_none_^c^			PD	BP_extreme_^a^	BP_mild_^b^	BP_none_^c^		
	*n* = 267	*n* = 79	*n* = 86	*n* = 102	*P*-value	Posthoc^d^	*n* = 237	*n* = 121	*n* = 38	*n* = 78	*P*-value	Posthoc^d^
SH, n (%)	23 (8.6)	23 (29.1)	—	—			16 (6.8)	16 (13.2)	—	—		
OH, n (%)	48 (18.0)	48 (60.8)	—	—			77 (32.5)	77 (63.6)	—	—		
∆HR/∆SBP_***3min***_ < 0.5, n (%)	46 (95.8)	—	—	—			74 (96.1)	—	—	—		
SH + OH, n (%)	3 (1.1)	3 (3.8)	—	—			20 (8.4)	20 (16.5)	—	—		
dOH, n (%)	22 (8.2)	—	22 (25.6)	—			14 (5.9)	—	14 (36.8)	—		
OHT, n (%)	64 (24.0)	—	64 (74.4)	—			24 (10.1)	—	24 (63.2)	—		
SH+dOH, n (%)	0 (0.0)	—	—	—			2 (0.8)	2 (1.7)	—	—		
SH + OHT, n (%)	5 (1.9)	5 (6.3)	—	—			6 (2.5)	6 (5.0)	—	—		
Supine SBP, mmHg	123.5 ± 15.2	134.3 ± 17.5	120.3 ± 11.5	117.8 ± 11.2	<0.001	a > b***, a > c***	126.6 ± 17.7	127.5 ± 16.4	120.0 ± 13.2	119.1 ± 13.8	<0.001	a > b*, a > c***
Supine DBP, mmHg	71.0 ± 8.6	75.0 ± 9.2	70.6 ± 8.6	68.2 ± 6.8	<0.001	a > b**, a > c***	71.9 ± 9.1	71.9 ± 8.9	70.1 ± 9.7	70.1 ± 7.9	0.293	
Supine HR, bpm	65.4 ± 9.6	66.3 ± 10.1	66.3 ± 9.6	64.0 ± 9.1	0.167		66.2 ± 13.6	65.5 ± 10.4	65.2 ± 10.0	65.6 ± 9.1	0.980	
orthostatic ΔSBP, mmHg	9.6 ± 12.6	21.2 ± 14.0	3.0 ± 8.9	6.0 ± 6.7	<0.001	a > b***, a > c***,b < c*	17.5 ± 16.4	14.8 ± 13.5	3.1 ± 8.2	4.3 ± 9.3	<0.001	a > b***, a > c***
orthostatic ΔDBP, mmHg	2.4 ± 7.7	9.0 ± 8.7	−1.8 ± 6.1	0.7 ± 3.8	<0.001	a > b***, a > c***,b < c**	6.3 ± 9.5	5.1 ± 8.1	−0.5 ± 5.9	−0.1 ± 6.5	<0.001	a > b***, a > c***
orthostatic ΔdSBP, mmHg	11.1 ± 15.9	22.9 ± 21.0	4.3 ± 11.8	7.5 ± 6.8	<0.001	a > b***, a > c***	18.7 ± 20.4	15.8 ± 18.1	5.9 ± 9.7	6.9 ± 14.5	<0.001	a > b**, a > c***
orthostatic ΔdDBP, mmHg	2.8 ± 9.8	9.0 ± 12.5	−0.8 ± 9.0	1.2 ± 4.3	<0.001	a > b***, a > c***	7.0 ± 12.3	5.2 ± 10.6	0.0 ± 8.4	1.0 ± 9.2	<0.01	a > b*, a > c*
orthostatic ΔSBP_***max,***_ mmHg	0.1 ± 13.2	10.4 ± 14.7	−7.7 ± 11.2	−1.2 ± 6.7	<0.001	a > b***, a > c***,b < c***	7.0 ± 16.0	4.7 ± 15.0	−5.8 ± 10.6	−3.8 ± 9.4	<0.001	a > b***, a > c***
orthostatic ΔDBP_***max,***_ mmHg	−4.6 ± 7.3	0.6 ± 7.6	−9.7 ± 6.8	−4.4 ± 3.3	<0.001	a > b***, a > c***,b < c***	−0.7 ± 8.7	−2.2 ± 8.0	−7.5 ± 6.2	−6.7 ± 5.7	<0.001	a > b***, a > c***
Early H/M ratio	1.57 ± 0.32	1.50 ± 0.31	1.63 ± 0.34	1.57 ± 0.30	0.029	a < b*	1.46 ± 0.27	1.42 ± 0.26	1.52 ± 0.26	1.51 ± 0.29	0.031	++
Delay H/M ratio	1.54 ± 0.39	1.45 ± 0.37	1.62 ± 0.41	1.54 ± 0.37	0.024	a < b*	1.43 ± 0.33	1.36 ± 0.29	1.48 ± 0.34	1.51 ± 0.37	0.008	a < c*
Washout H/M rate, %	2.51 ± 8.79	3.32 ± 9.01	1.43 ± 8.11	2.80 ± 9.16	0.355		2.87 ± 8.83	4.17 ± 7.90	3.14 ± 10.92	0.73 ± 8.79	0.026	a > c*

Data are shown as mean ± standard deviation unless remarked otherwise. One-way analysis of variance (ANOVA) or Kruskal-Wallis tests were performed for continuous or ordinal variables, and Fisher’s exact test was used for categorical variables to compare between-group differences. *BP*_*extreme*_ extreme BP dysregulation group, *BP*_*mild*_ mild BP dysregulation group, *BP*_*none*_ no BP dysregulation group, *SH* supine hypertension, *OH* orthostatic hypotension, *dOH* delayed OH, *OHT* orthostatic hypertension, *SBP* systolic blood pressure, *DBP* diastolic blood pressure, *dSBP* delayed systolic blood pressure, *dDBP* delayed diastolic blood pressure, *HR* heart rate, *H/M* heart-to-mediastinum, bpm beat-per-minute.

^d^Multiple comparisons were adjusted by Tukey, Games-Howell, or Dwass-Steel-Critchlow-Fligner methods, as appropriate.

+Follow-up loss (*n* = 30) is not shown in the table.

+ +Pairwise comparisons did not survive multiple comparison adjustments.

*p < 0.05; **p < 0.01; ***p < 0.001.

At initial evaluation, 23 PD patients (8.6%) were hypertensive in the supine position; 48 (18.0%) patients were OH. Twenty-two (8.2%) were determined to have delayed OH, and 64 (24.0%) had orthostatic hypertension. The two OH groups were not different in age at diagnosis, disease duration, or H/M ratio (Supplementary Table 1). The prevalence of OH increased during the follow-up examination period. More than 95% of PD patients with OH experienced reduced cardiac baroreflex gain, indicating its neurogenic origin (initial *vs*. follow-up ∆HR/∆SBP_3min_ < 0.5, 95.8% vs. 96.1%).

The initial converted MDS-UPDRS Part II score was 6.6 ± 4.3, and this did not differ among the orthostatic subgroups. This lack of difference remained at follow-up between-group comparisons. The initial motor score, part III, was 18.5 ± 9.1, and its severity was similar between groups. This score increased to 19.5 ± 11.0 at the follow-up exam, and the BP_extreme_ group had more severe motor impairment than the BP_none_ group (21.1 ± 12.2 vs. 17.5 ± 7.6). The median H&Y stage was two; this grade was maintained throughout the follow-up. PIGD and AR phenotypes were the dominant forms. The motor phenotype was significantly associated with the initial orthostatic subtype, particularly BP_extreme_ related to PIGD (TD´ vs. PIGD vs. intermediate, 16.5% vs. 69.6% vs. 13.9%).

**Fig. 1:**
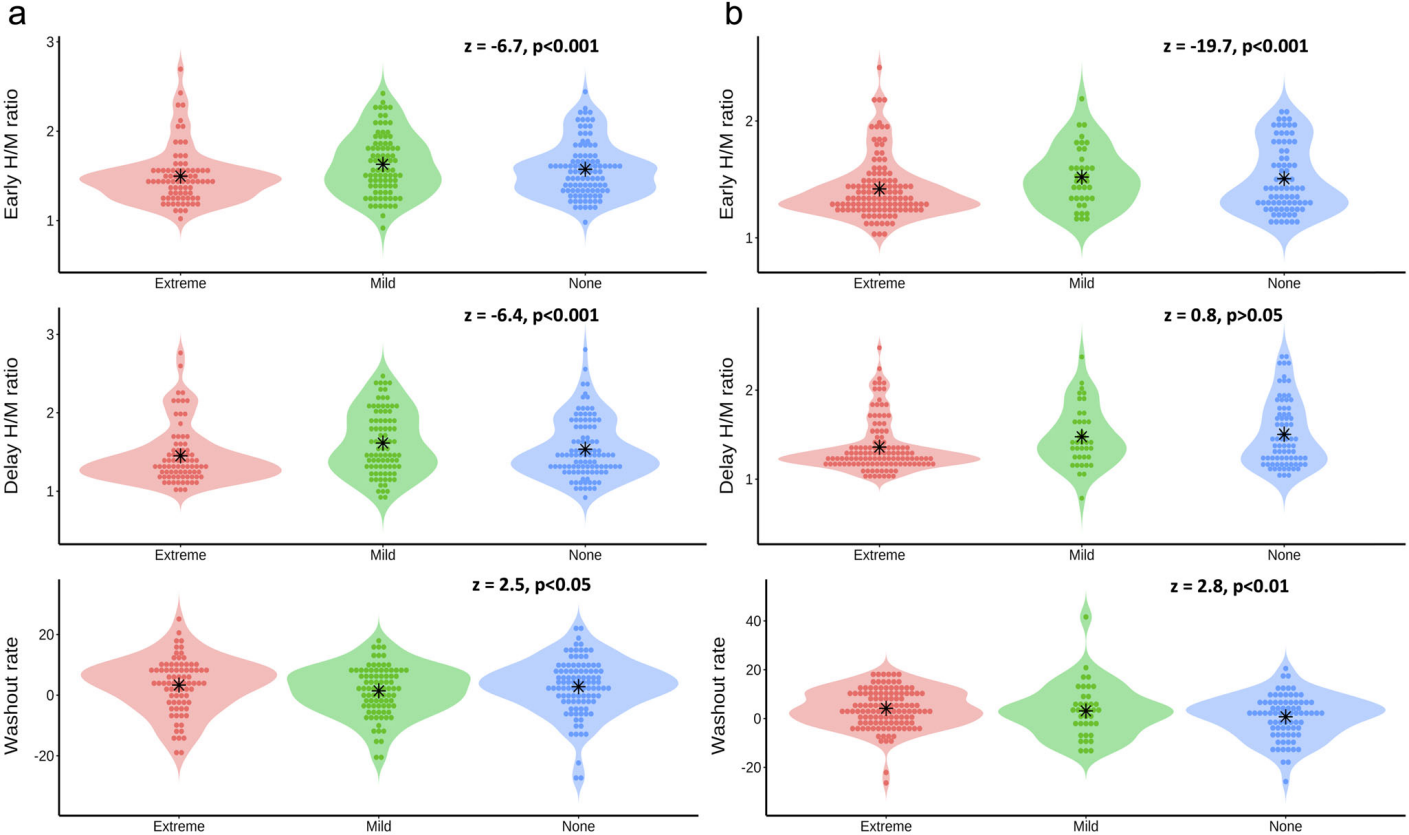
The linear trend of cardiac sympathetic denervation across orthostatic subtypes at each time point. **a**, **b** represent the linear trend of cardiac denervation at initial (*n* = 267) and follow-up (*n* = 237) populations, respectively. A negative z value in Jonckheere-Terpstra test indicates an ascending linear trend, whereas a positive value represents a descending linear trend across groups. Black stars mark the central tendency of each subgroup distribution. H/M heart-to-mediastinum.

The overall H/M uptake ratios for both early and delayed groups were below the pre-defined reference. The BP_extreme_ group had the smallest tracer uptake ratio overall and the highest washout rate (WR). Trend analyses of the tracer uptake ratio revealed a gradient of increase or decrease in the groups (Fig. 1). Early and delayed H/M ratio demonstrated a linear increase, while the WR showed decremental linearity across subtypes (BP_extreme_ vs. BP_mild_ vs. BP_none_).

### Overall changes of orthostatic subtypes and motor phenotypes over time

**Fig. 2:**
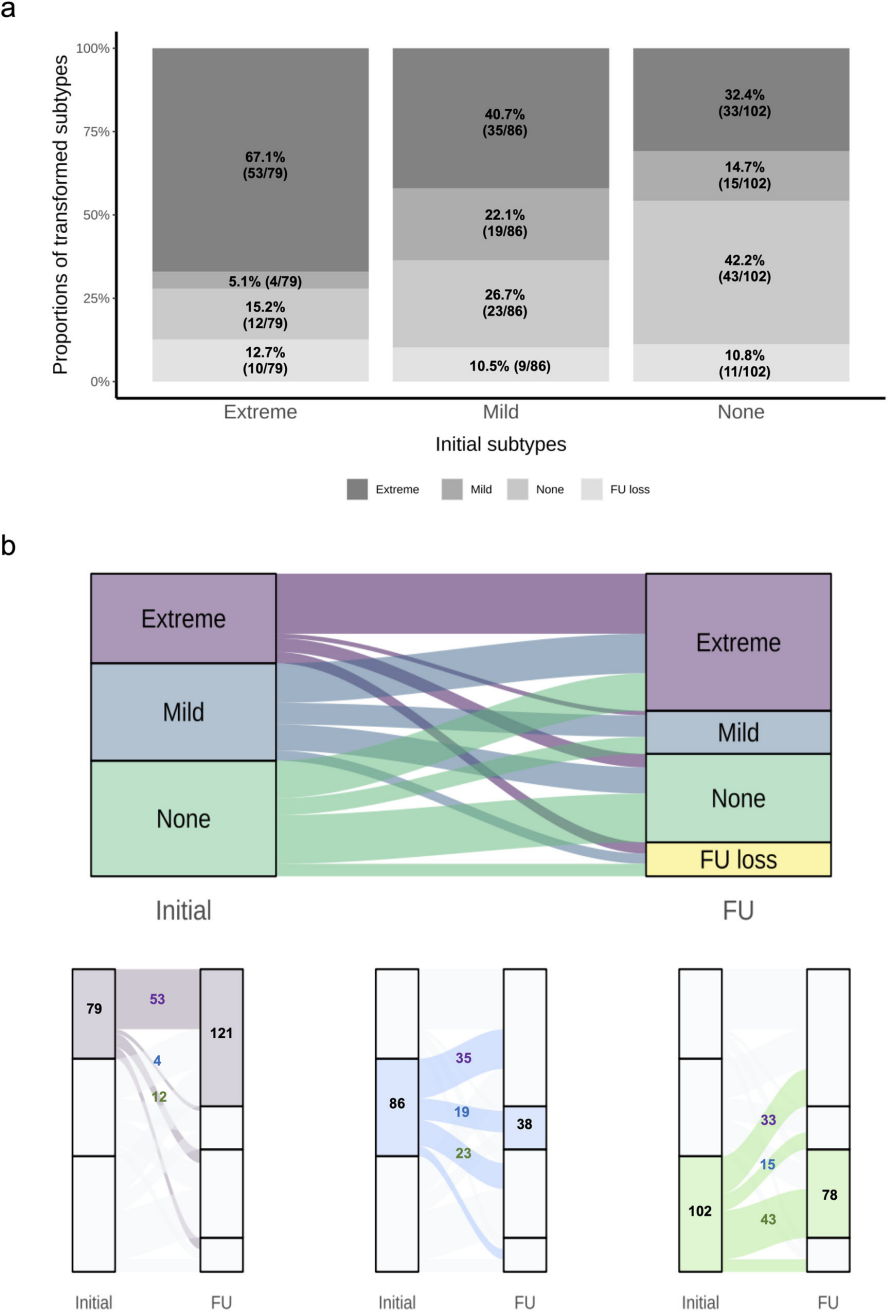
The prevalence of orthostatic blood pressure dysregulation patterns over time. **a** The prevalence of converted subtypes among initial orthostatic subtypes, and **b** flow diagram of orthostatic subtype conversions are demonstrated. The numbers indicate the number of patients in that group, and colored numbers within the flows represent the converting patients between the alluviums.

**Fig. 3:**
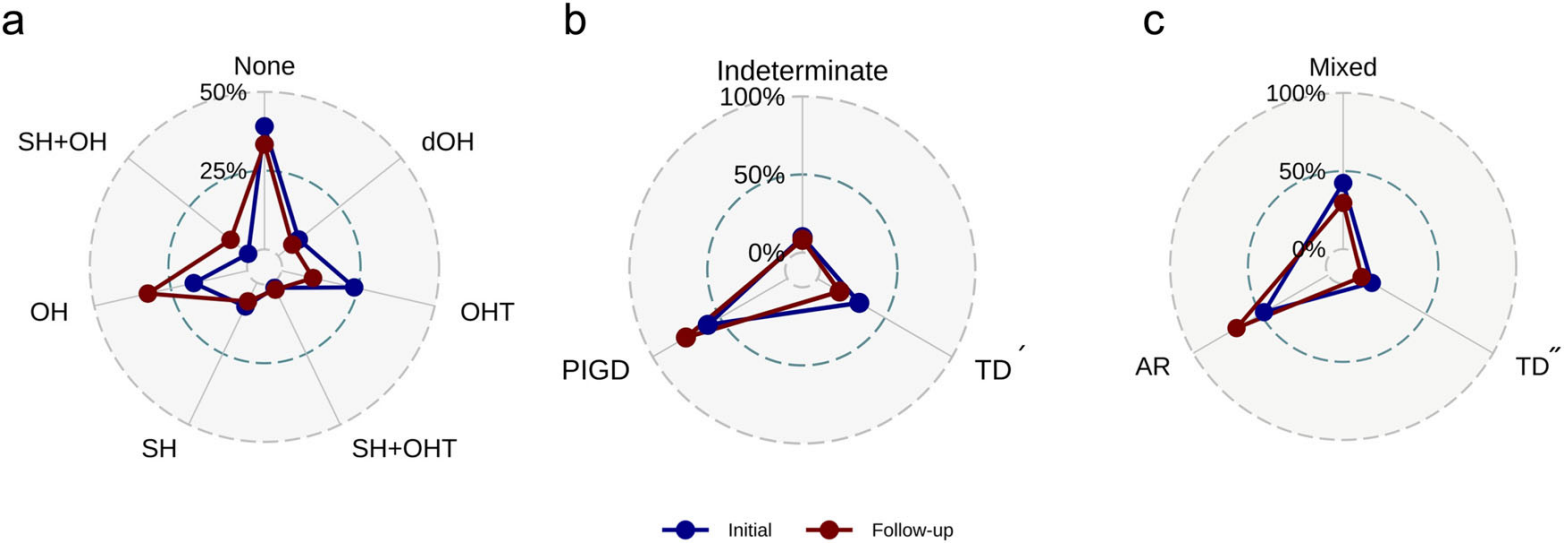
The prevalence changes in orthostatic and motor subtypes across time. **a** represents changes in orthostatic subtype; **b** motor PIGD versus TD phenotype; and **c** motor AR versus TD phenotype. Two SH+dOH patients at the follow-up are not represented in the figure because its subtype did not exist at the initial; thus, its changes could not be plotted in the radar chart. dOH delayed orthostatic hypotension, OHT orthostatic hypertension, SH supine hypertension, OH orthostatic hypotension, TD tremor dominant, PIGD postural instability/gait difficulty, AR akinetic-rigid.

Positional ∆BP types progressed in different distribution patterns after 29.3 ± 9.4 months (Figs. 2 and 3). The BP_extreme_ group (especially OH and SH + OH) increased, while patients with BP_mild_ group decreased. About one-third of patients in the initial BP_extreme_ group changed to an orthostatic BP dysregulation subtype, while the rest retained their subtype at follow-up (67.1% of the initial status; Fig. 2a, b). Among the BP_mild_ patients, 40.7% progressed to BP_extreme_, while 22.1% remained as the initial subtype (Fig. 2a, b). Of the initial BP_none_ group, 32.4% and 14.7% progressed to orthostatic dysregulation (BP_extreme_ vs. BP_mild_; respectively), while 42.2% remained free of orthostatic stress (Fig. 2a, b). At the follow-up, a total of 121 participants were classified as BP_extreme_ group (fifty-three from initial BP_extreme_; thirty-five from initial BP_mild_; thirty-three from initial BP_none_). A linear ascending trend of delayed H/M at both initial and follow-up was observed across turnover patterns of BP_none_ subtype (initial BP_subtype_ → follow-up BP_subtype_; BP_none_ →BP_extreme_ vs. BP_none_ →BP_mild_ vs. BP_none_ →BP_none_; Supplementary Fig. 2). The proportions of those either transforming into or maintaining BP_none_ group increased across the initial subtypes (initial BP_subtype_ → follow-up BP_subtype_; BP_extreme_ →BP_none_ vs. BP_mild_ →BP_none_ vs. BP_none_ →BP_none_; 15.2% vs. 26.7% vs. 42.2%; Cochran-Armitage test, *χ*^2^_(1)_ = 15.9, *p* < 0.001, Fig. 2a).

The distributions of motor phenotypes also changed (Fig. 3). The PIGD prevalence increased, while that of TD´ decreased; AR type increased as TD˝ and mixed types decreased.

### Sub-analyses of orthostatic subtype transformation pattern

Initial BP_mild_ and BP_none_ groups were selected for sub-analyses to compare the characteristics before and after subtype transformation (Table 3). No discernible between-group differences were observed except for patients with BP_mild_, among whom those with status improvement to normal orthostatic response (BP_mild_→BP_none_) showed shorter disease duration before diagnosis. There were no associations between motor phenotypes and subtype transformation patterns.

**Table 3:** Subgroup analysis of initial subtype changes across time.

BP_mild_ (*n* = 86), initial	BP_mild_ → BP_extreme_^a^	BP_mild_ → BP_mild_^b^	BP_mild_ → BP_none_^c^		
	*n* = 35	*n* = 19	*n* = 23	*P*-value	Posthoc^d^
Age at diagnosis, years	65.8 ± 7.6	65.5 ± 10.2	62.3 ± 10.5	0.332	
Disease duration at diagnosis, months	16.3 ± 14.7	13.5 ± 10.7	8.8 ± 5.9	0.018	a > c*
^123^I-MIBG Follow-up interval, months	30.6 ± 11.2	31.1 ± 11.3	31.3 ± 9.8	0.970	
Early H/M_***i***_ ratio	1.55 ± 0.36	1.72 ± 0.29	1.69 ± 0.33	0.144	
Delay H/M_***i***_ ratio	1.52 ± 0.42	1.74 ± 0.38	1.67 ± 0.37	0.126	
Δ Early H/M ratio	5.6 ± 12.4	11.1 ± 11.4	9.6 ± 14.6	0.276	
Δ Delay H/M ratio	7.2 ± 12.1	12.1 ± 14.4	9.0 ± 17.7	0.502	
Motor phenotype´				0.385	
TD´, *n* (%)	8 (22.9)	6 (31.6)	8 (34.8)		
PIGD, *n* (%)	25 (71.4)	13 (68.4)	12 (52.2)		
Intermediate, *n* (%)	2 (5.7)	0 (0.0)	3 (13.0)		
Motor phenotype˝				0.380	
TD˝, *n* (%)	0 (0.0)	1 (5.3)	2 (8.7)		
AR, *n* (%)	24 (68.6)	11 (57.9)	12 (52.2)		
Mixed, *n* (%)	11 (31.4)	7 (36.8)	9 (39.1)		
LEDD, mg	474.2 ± 199.8	442.0 ± 130.7	401.1 ± 183.9	0.324	

BP_none_ (*n* = 102), initial	BP_none_ → BP_extreme_^a^	BP_none_ → BP_mild_^b^	BP_none_ → BP_none_^c^		
	*n* = 33	*n* = 15	*n* = 43	*P*-value	Posthoc^d^
Age at diagnosis, years	67.4 ± 7.3	62.0 ± 12.3	66.2 ± 9.4	0.300	
Disease duration at diagnosis, months	14.0 ± 11.0	14.7 ± 7.2	11.4 ± 10.7	0.407	
^123^I-MIBG Follow-up interval, months	29.5 ± 9.1	28.8 ± 7.7	29.2 ± 9.1	0.972	
Early H/M_***i***_ ratio	1.51 ± 0.26	1.60 ± 0.28	1.62 ± 0.32	0.282	
Delay H/M_***i***_ ratio	1.43 ± 0.32	1.57 ± 0.32	1.61 ± 0.40	0.091	
Δ Early H/M ratio	5.4 ± 10.4	2.1 ± 13.4	6.0 ± 11.6	0.526	
Δ Delay H/M ratio	5.1 ± 12.5	0.6 ± 16.3	4.4 ± 13.3	0.551	
Motor phenotype´				0.969	
TD´, *n* (%)	3 (9.1)	2 (13.3)	6 (14.0)		
PIGD, *n* (%)	27 (81.8)	12 (80.0)	33 (76.7)		
Intermediate, *n* (%)	3 (9.1)	1 (6.7)	4 (9.3)		
Motor phenotype˝				0.122	
TD˝	0 (0.0)	0 (0.0)	2 (4.7)		
AR	21 (63.6)	9 (60.0)	34 (79.1)		
Mixed	12 (36.4)	6 (40.0)	7 (16.3)		
LEDD, mg	431.3 ± 159.6	479.7 ± 257.6	475.8 ± 312.1	0.719	

Data are shown as mean ± standard deviation unless remarked otherwise. One-way analysis of variance test for continuous variables and Fisher’s exact test for categorical variables were performed compare between-group differences. Follow-up losses are not shown in the table. *BP*_*extreme*_ extreme BP dysregulation group, *BP*_*mild*_ mild BP dysregulation group, *BP*_*none*_ no BP dysregulation group, ^*123*^*I-MIBG* ^123^I-meta-iodobenzylguanidine, *LEDD* levodopa equivalent daily dose, subfix i, initial.

^d^Multiple comparisons were adjusted by Tukey or Games-Howell methods, as appropriate.

*p < 0.05.

## Discussion

Early PD, the diagnosis of which was re-affirmed at an average follow-up of five years, was investigated for positional adrenergic failure. Cardiovascular dysautonomia was sub-grouped into BP_extreme_ (SH and/or OH with any other forms), BP_mild_ (dOH or OHT), and BP_none_ (PD without orthostatic BP dysregulation). The BP_extreme_ group was initially associated with PIGD but lost its relationship during follow-up. The number of patients in the BP_extreme_ group increased, while that of those with BP_mild_ decreased after 2–3 years. A large proportion of the BP_mild_ group progressed to BP_extreme_. The transformations of orthostatic subtypes suggested a dynamic classification along its adrenergic failure: BP_none_ → BP_mild_ → BP_extreme_.

In this study, the PD population was in the early stage of disease at enrollment. The patients were at the mid-phase of the disease when their diagnosis was reviewed for the study. The point prevalence of PD with OH (PD + OH) at initial presentation and follow-up was comparable to that in a previous study by Hiorth et al.^13^. That group estimated the point prevalence at the final visit to be 32.8%, but the cumulative incidence of OH was centered at a median of 1.7 years (interquartile range, 1.0–2.5 years). Therefore, most newly detected OH occurred within 2.5 years and then approached a plateau. This approximates our 32.5% prevalence at an average of 29.3 ± 9.4 months. The age of the BP_extreme_ group was the oldest among the groups. Previous studies reported the OH group to be the oldest; this difference could be explained by the status of OH as the major constituent of the BP_extreme_ group^5,13^.

The prevalence of SH and SH + OH was lower in the present study than in previous estimates^2,14–16^. This difference could be attributed to how supine BP was calculated. This study used the average of four separate measurements, which might have resulted in a lower estimate of SH. The study focused on accurately identifying SH, as it can co-occur with neurogenic OH in PD^2^, making its separation crucial for the study’s orthostatic subtyping. The definitions of OH and OHT were influenced by supine BP, which served as a reference for comparing standing BP. The study aimed to provide a conservative estimation of the prevalence of different subtypes, leading to a lower prevalence of SH. However, the baseline prevalence of OHT in this study was similar to that of other previous work^4^.

PD Patients with SH and/or OH (BP_extreme_) represent the extremes of hemodynamic adrenergic failure in its respective supine and upright positions by a potentially shared pathophysiology^7^. Delayed OH and OHT (BP_mild_) previously were conceptualized to be situated between PD without cardiovascular dysautonomia (BP_none_) and BP_extreme_ along a spectrum of adrenergic failure^3–5,8^. The characteristics of delayed OH and OHT also did not differ in the present study; thus, they were grouped to represent BP_mild_. The H/M uptake ratios and washout rates reflected postganglionic cardiac sympathetic denervation and its progressing catecholaminergic malfunction^17–19^. Trend analyses revealed increasing uptake ratios and decreasing washout rates, implying a gradient of adrenergic failure across the groups. Higher H/M uptake ratios and lower washout rates correlated with more preserved sympathetic integrity. These gradients persisted during follow-up. The initial establishment and maintenance of the adrenergic gradient during disease progression support the argument of BP_mild_ as an intermediary phase, indicating possible temporal evolution among the subtypes (BP_none_ *→* BP_mild_ *→* BP_extreme_).

The BP_extreme_ group was assumed to have the most severe adrenergic failures and increased in number over time; while the BP_mild_ group, presumably the lesser impaired, decreased. Most patients with initial BP_extreme_ maintained in the extreme group, while many in the BP_mild_ group deteriorated into BP_extreme_. This stepwise deterioration was presumed to occur because the extreme subclass lacked the reserve for progression while the milder forms allowed remnant biology to progress. In a sub-analysis of turnover patterns of BP_none_ to other subtypes, a linear gradient indicated that BP_none_*→*BP_extreme_ (the most severe form) was correlated with the most impaired sympathetic tone. In contrast, the BP_none_*→*BP_none_ (the most preserved form) demonstrated the least impairment. This association between the severity of sympathetic tone and the grade of orthostatic turnovers could provide another explanation of the underlining pathophysiologic nature of BP dysregulations (BP_none_ *→* BP_mild_ *→* BP_extreme_).

The proportion of patients resolving to BP_none_ increased significantly across the initial subtypes. This implies that increasing remnant sympathetic function, as indicated by increased linear H/M uptake ratio, played a role in such transformation. Within the context of PD as a neurocardiologic disease, we interpreted regression to BP_none_ to be a result of different resiliencies of extant sympathetic functions among subtypes, rather than actual resolution^6^. Some of the transformations into normal BP regulation might result from the hypotensive effect of L-DOPA, particularly in the SH + OHT group (BP_extreme_) which needs further clarification with a larger population in future research.

The non-tremor-dominant type was the most prevalent motor phenotype in this cohort despite the absence of a significant difference. Both PIGD and AR types increased during follow-up, while others decreased. The most impaired subtype, BP_extreme_, showed the largest overlap with the PIGD or AR motor phenotype. Motor impairment was greater in this extreme group than in the others at follow-up. We presumed that this was not a coincidence since BP_extreme_ and non-tremor-dominant PD correlated with poor outcomes^6,7,9,20,21^, and BP_extreme_ was suggested to demonstrate diffuse pathology^22^. Future longitudinal studies are warranted to assess the association between motor prognosis and orthostatic subtype.

This study has some strengths. First, we attempted to reduce selection bias by including PD patients who were re-assessed after at least two years and clinically followed for an average of five years. Another strength was the distinction of the OHT group, allowing for a hypertensive span in both supine and upright positions. The inclusion of an OHT group is rare in descriptions of cardiovascular dysautonomia in patients with PD, and the present study provides a baseline for future studies. In addition, various labile BP subgroups were studied. Finally, this study demonstrated the change in each subtype from baseline, allowing observation of the dynamic changes of BP dysregulations.

However, this study also had several limitations. One limitation is that the follow-up head-up tilt test period was relatively short. One purpose of this study was to investigate whether transformation between orthostatic subtypes could indicate the severity of neurobiology, avoiding the influence of confounders such as the administration of dopaminergic medications. Had the study design included results of patients with advanced PD, the changes of subtypes could have been significantly affected by the effects of medication. However, prolonged follow-up, including those in the advanced stage would be informative since there lacks of prospective investigation of the prevalence of diverse orthostatic subtypes that reflect clinical reality. The exclusion criteria also could have biased the results as it might prohibit the enrollment of PD patients who indeed had orthostatic adrenergic failure, regardless of confounders. In a study looking into the natural course of a disease, the excluded patients could provide precious information that mirrors the clinical circumstances; thus, offering generalizability in its interpretation. Another limitation is that clinical severity was not related to laboratory parameters. The study would be more complete if the longitudinal clinical symptoms, other than motor phenotypes, were associated with longitudinal changes. Future study encompassing both clinometric and laboratory findings is needed. Lastly, we did not include any suspected cases with clinical manifestations of atypical PD in the study. We investigated only PD patients whose diagnosis was assured by the two neurologists. Their diagnoses were confirmed by reviewing the patients’ clinical course after an average follow-up period of 61.4 ± 21.8 months. This signified that only clinically confirmed PD patients with much confidence were enrolled initially, and their *early* clinical course of the total follow-up (approximately the first one-third of the disease duration) was investigated. This *retrospectively confirmed prospective nature* of the study design prevented the authors from estimating the transition to atypical PD, such as multiple system atrophy, because they were not considered during clinical retrospection. The experimental design represents one major bias, but in our belief, it is also a strength because no single biomarker could replace the clinical follow-ups of neurologists in confirming PD. Although more extended periods of follow-ups are necessary, the average total follow-up duration allowed the neurologists to assert their re-affirmation of PD. Future prospective research, inclusive of all parkinsonism, is warranted to assess the development of multiple system atrophy.

In conclusion, various forms of positional BP dysregulation existed in the early PD patients in this study. Although the exact pathobiology of each type and their interrelationships are not fully understood, OHT and delayed OH might be a preliminary phase before progressing to SH and OH. Orthostatic subtypes might represent the severity of adrenergic dysfunction in early PD regardless of dopaminergic medication use.

## Methods

### Patients

This study was approved by the Institutional Review Board of Seoul St. Mary’s Hospital. All subjects provided written informed consent to participate. The research was conducted in accordance with relevant guidelines and regulations.

Two hundred sixty-seven drug-naive and de novo PD patients presenting between August 2012 and July 2020 were enrolled in this cohort. PD was diagnosed based on criteria of the UK Parkinson’s Disease Society Brain Bank^23^, and its diagnosis was supported by positron emission tomography studies using ^18^F-N-(3-fluoropropyl)-2beta-carbon ethoxy-3beta-(4-iodophenyl) nortropane^24^. Patients showed decreased dopamine transporter uptake in the striatum, primarily in the posterior putamen. Patients were monitored every 2–6 months for at least 24 months and a maximum of 10 years (average follow-up duration, 61.4 ± 21.8 months, Supplementary Fig. 1). Diagnoses were confirmed during follow-up by two neurologists, S.-W.Y. and J.-S.K. Thirty patients were excluded from the study because they were lost to the follow-up examinations.

Baseline characteristics of age; sex; body mass index; disease duration at diagnosis; follow-up duration; history of hypertension, diabetes mellitus, or dyslipidemia; and smoking status were investigated at the initial assessment. Patients with any of the following were excluded: (1) any symptoms or signs of atypical and/or secondary parkinsonism, (2) family history of PD among first-degree relatives, (3) history of diabetic neuropathy, (4) history of symptomatic stroke that could affect general cognition and performance, (5) history of heart failure that required cardinal symptoms and signs of exercise intolerance (such as exertional dyspnea) and fluid retention (such as edema)^25^, and (6) current intake of medications known to influence autonomic functions, such as alpha-blockers or tricyclic antidepressants.

### UPDRS and MDS-UPDRS

The 267 patients were initially evaluated using the Unified Parkinson’s Disease Rating Scale (UPDRS), and ten were also assessed using the Movement Disorder Society (MDS)-UPDRS. At the follow-up examinations (*n* = 237), 175 were assessed using the UPDRS; the remainder were evaluated using the MDS-UPDRS. The Hoehn and Yahr (H&Y) stage was scored at every assessment. The subtotal scores of parts II and III of the UPDRS were scaled into the MDS-UPDRS for comparisons^26^.

Two motor phenotyping methods were applied to stratify the PD patients. These methods were tremor dominant (TD´) versus postural instability/gait difficulty (PIGD) or tremor dominant (TD˝) versus akinetic-rigid (AR) type^27–29^. The motor phenotypes were determined by the original scales, either UPDRS or MDS-UPDRS (Supplementary Method).

The average examination interval was 29.5 ± 9.7 months (Supplementary Fig. 1).

### Head-up tilt-test

Head-up tilt-test was performed after discontinuing any antihypertensive medications for at least 7 days. Patients were also asked to abstain from drinking alcohol or coffee the day before the test. All patients were tested in the full resting state. Continuous electrocardiograph leads and non-invasive BP monitoring equipment were applied to the patients (YM6000, Mediana Tech, Redoman, WA, USA). After 20 min of supine rest, head-up tilt testing (20 min at 60°) was performed using a Manumed Special Tilt1-section tilt table (ENRAF NONIUS, Rotterdam, The Netherlands). Supine BP and heart rate were recorded every 5 min before tilting to 60°, and the same measurements were performed at 0, 3, 5, 10, 15, and 20 min during head-up tilt, and as deemed necessary to ensure subject safety.

After excluding the first supine BP (at 0 min), average supine systolic/diastolic BPs and heart rates (HR; beats per minute) were calculated from the measurements at 5, 10, 15, and 20 min. Supine hypertension (SH) was diagnosed if the average supine systolic and/or diastolic BP (SBP/DBP) was ≥ 140/90 mmHg^30^.

The lowest SBP/DBP (BP_min_) at three or five minutes in the tilted position was selected to identify orthostatic hypotension (OH). The orthostatic BP decreases in systole (ΔSBP) and diastole (ΔDBP) were calculated from average supine BPs. When patients were supine-hypertensive, ΔSBP and/or ΔDBP ≥ 30/15 mmHg within five minutes was used to diagnose OH; otherwise, ΔSBP and/or ΔDBP ≥ 20/10 mmHg were used^4,30,31^. Patients were classified as delayed OH (dOH) when they met the above criteria after 10 min.

The orthostatic ΔBP was re-calculated from the highest BP (BP_max_) among the tilted measures at 3, 5, 10, and 15 min (average supine BP minus highest orthostatic BP_max_). Patients with PD with SH were described as having orthostatic hypertension (OHT) if ΔSBP_max_ and/or ΔDBP_max_ was ≤−20/10 mmHg. Patients with PD without SH were categorized as OHT when orthostatic BP_max_ was ≥140/90 mmHg or ΔBP_max_ was ≤−20/10 mmHg^4,32^. Positive ΔSBP_max_ and/or ΔDBP_max_ signified a decrease in upright ΔBP, and negative values indicated an increase in orthostatic ΔBP.

OH, dOH, and OHT diagnoses were mutually exclusive; only SH could be co-diagnosed with any of these three subtypes. For example, PD with OH patients could not be classified as dOH or OHT but could be subtyped into SH + OH.

PD patients with SH and/or OH in any combination with other forms (SH, OH, SH + OH, SH+dOH, SH + OHT) were classified as BP_extreme_, and PD with dOH or OHT was assigned as BP_mild_. If SH co-existed with either dOH or OHT, the patient was classified as BP_extreme_. PD with no orthostatic subtype was categorized as BP_none_. These subgroups were designed to represent the grades of adrenergic failure.

Orthostatic HR at three minutes was selected to estimate the change in HR (ΔHR) from average supine HR, and its values were utilized to calculate ΔHR/ΔSBP at three minutes (ΔHR/ΔSBP_3min_)^33,34^. The measurement was only applied to those with PD with OH as the original description.

The average interval between investigations was 29.3 ± 9.4 months (Supplementary Fig. 1).

### Levodopa equivalent daily dose (LEDD; mg)

The most recent dopaminergic dosage at the time of head-up tilt test was calculated as levodopa equivalent daily dose^35^.

### ^123^I-metaiodobenzylguanidine (^123^I-MIBG) myocardial scintigraphy

^123^I-MIBG scintigraphy was performed using a dual-head camera equipped with a low-energy, high-resolution collimator (Siemens), and data were collected at 30 minutes (early) and 120 min (delayed) after injection of 111 MBq of ^123^I-MIBG. A static image was obtained with a 128 × 128 matrix. Regions of interest (ROIs) were drawn manually around the heart and mediastinum. Tracer uptake was measured within each ROI to calculate the heart-to-mediastinum (H/M) ratio for early and delayed time points. The washout rate (WR) was calculated as [(early H/M ratio – late H/M ratio)/early H/M ratio] × 100. Reference values for normal H/M ratio were 1.70 for early and 1.78 for delayed ratios^36^.

Patients were evaluated initially at the time of diagnosis, and the changes of tracer uptake were re-assessed after 30.0 ± 9.6 months (Supplementary Fig. 1).

### Statistical analyses

Statistical analyses were conducted with jamovi software (version 2.3.18; retrieved from https://www.jamovi.org) for Mac, a graphical user interface for R. Analysis of variance (ANOVA) or Kruskal-Wallis test was performed for continuous or ordinal variables when appropriate. Categorical variables were examined by Fisher’s exact test. Cochran-Armitage test and Jonckheere-Terpstra test were executed in R (version 4.2.1; PMCMRplus package). Multiple comparisons were adjusted as appropriate. Statistical significance was defined as a two-tailed *p*-value < 0.05.

### Reporting summary

Further information on research design is available in the Nature Research Reporting Summary linked to this article.

## Supplementary information

Supplemental information

Reporting Summary

## Data Availability

Anonymized data generated during this study are available from the corresponding author on request from individuals affiliated with research or healthcare institutions.
