# Supplementary material for: A 3-year natural history of orthostatic blood pressure dysregulation in early Parkinson’s disease
Source: NPJ Parkinsons Dis. 2023 Jun 21;9:96. doi: 10.1038/s41531-023-00546-5 (PMC10284855; doi:10.1038/s41531-023-00546-5)
Supplement: Supplementary file 1 — Supplemental information [file 41531_2023_546_MOESM1_ESM.pdf]

Supplementary Figure 1. Flow diagram of enrolled patients and schematic representation of investigation

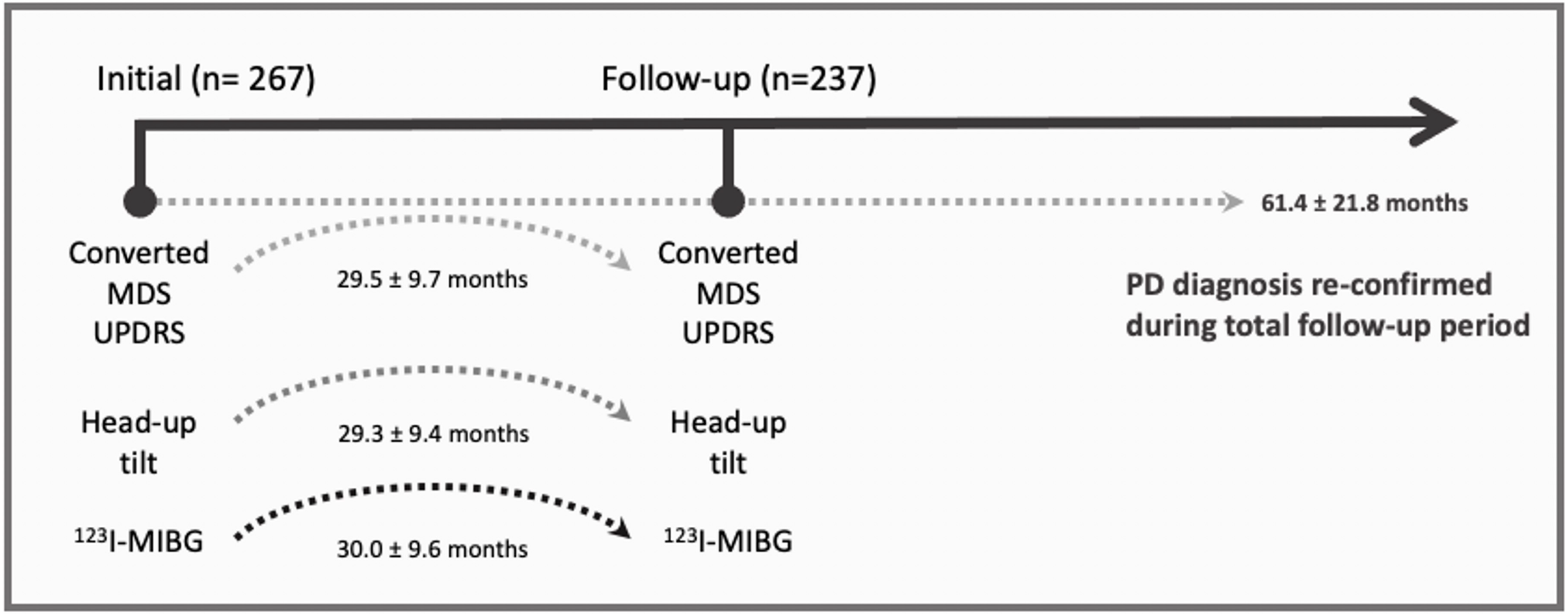

PD Parkinson's disease, MDS-UPDRS Movement Disorder Society- Unified Parkinson's disease Rating Scale, 123I-MIBG ^{123}I-metaiodobenzylguanidine scintigraphy

Supplementary Figure 2. Linear trend across orthostatic subtype changes.

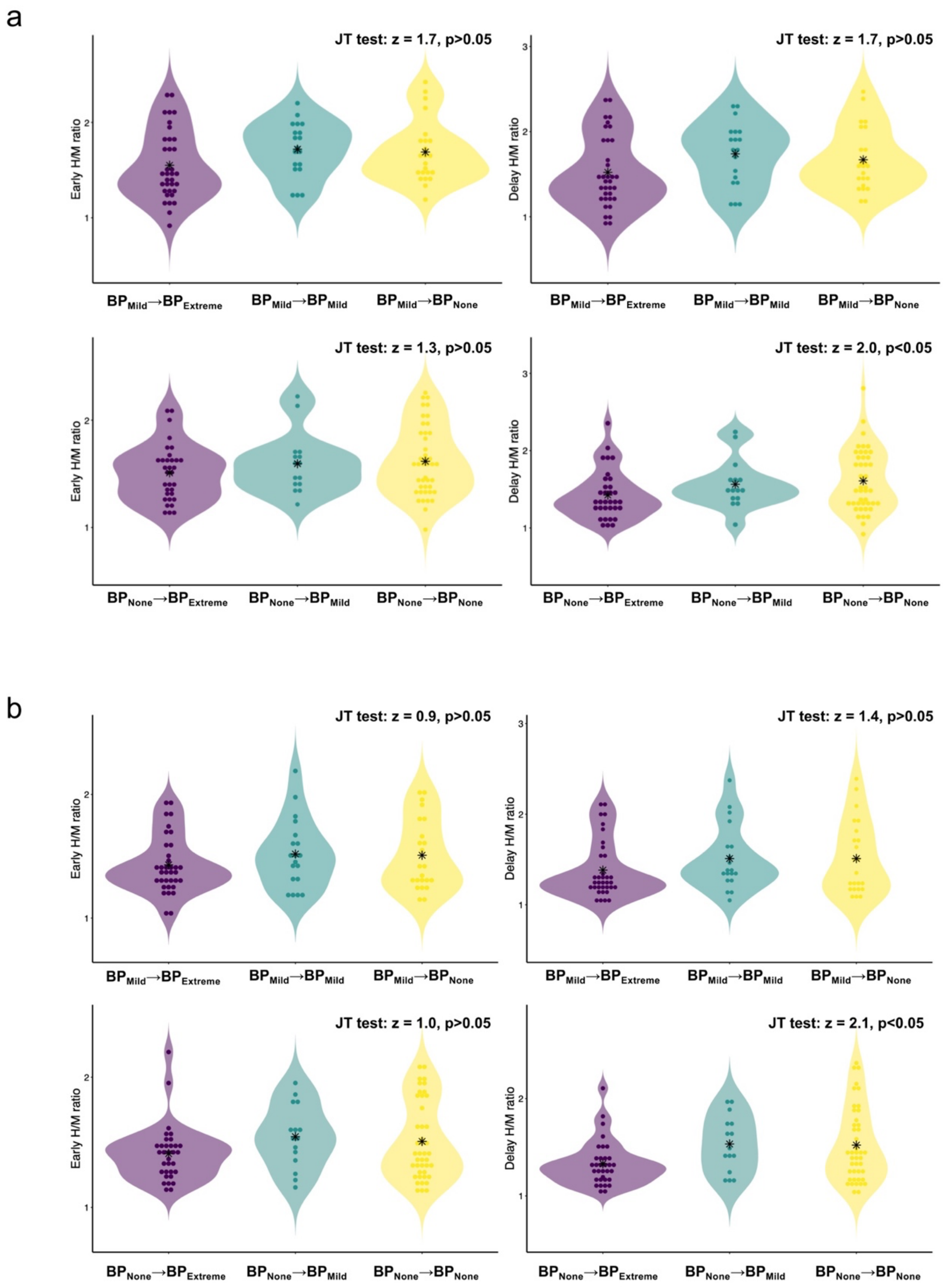

(a) represents the trend of initial heart-to-mediastinum ratio. (b) depicts the trend of the follow-up heart-to-mediastinum ratio. A positive z value represents an ascending linear trend. Black stars mark the central tendency of each subgroup distribution. JT test Jonckheere-Terpstra test

Supplementary Table 1. Between-group comparisons of delayed orthostatic hypotension and classic orthostatic hypertension at each time point.

	Initial (n=267)			Follow-up (n=237)		
	Delayed OH (n=22)	OH (n=64)	P-value	Delayed OH (n=14)	OH (n=24)	P-value
Age at diagnosis, years	63.9±7.7	65.1±9.7	0.616	67.6±10.0	62.2±11.3	0.150
Disease duration at diagnosis	16.1±13.6	12.8±11.1	0.263	17.0±11.8	14.1±10.0	0.421
^{123}I-MIBG Follow-up interval	-	-		32.9±13.0	29.8±8.2	0.373
Early H/M_i ratio	1.67±0.35	1.62±0.34	0.494	1.64±0.24	1.66±0.30	0.814
Delay H/M_i ratio	1.65±0.40	1.61±0.41	0.685	1.63±0.31	1.66±0.37	0.808
ΔEarly H/M ratio, %	8.7±13.7	7.9±12.8	0.819	6.8±11.8	7.1±13.3	0.944
ΔDelay H/M ratio, %	9.3±14.4	8.8±14.6	0.906	11.2±19.1	7.8±15.6	0.555
LEDD, mg	-	-		446.6±187.4	483.3±219.8	0.604

Data is shown as mean ± standard deviation unless remarked otherwise. Independent t-test was performed to compare between-group differences at each time point. Follow-up losses are not shown in the table. OH orthostatic hypotension, H/M heart-to-mediastinum, ^{123}I-MIBG ^{123}I-*meta*-iodobenzylguanidine, LEDD levodopa equivalent daily dose, subfix ***i*** initial.

Supplementary Methods

1. Tremor dominant (TD) vs. postural instability/gait difficulty (PIGD) subtypes

The motor phenotypes were determined according to relevant scales: items for tremor dominant (TD) *vs.* postural instability/gait difficulty (PIGD) subtypes for corresponding Unified Parkinson's Disease Rating Scale (UPDRS) and Movement Disorder Society-UPDRS (MDS-UPDRS) scores (Table 1).[1,2] Using the original UPDRS, the tremor dominant group was defined as those with a mean tremor score/mean PIGD score ≥ 1.5, and the PIGD group included all patients with a ratio ≤ 1.0. Ratio values between 1.0 and 1.5 were defined as the indeterminate subtype. In addition, the MDS-UPDRS method was used to calculate the ratio of the average tremor score to the average PIGD score. For this ratio, a value ≥ 1.15 was considered tremor dominant (TD′) subtype, and a value ≤ 0.9 was PIGD subtype; other values were indeterminate subtype. Patients who had a zero in the denominator were classified as TD, and those with a zero in the numerator and a positive mean in the denominator were PIGD subtype.

Table 1. The subscales of UPDRS and MDS-UPDRS for discrimination of tremor dominant vs. postural instability/gait difficulty (PIGD) subtypes.

UPDRS Unified Parkinson ' s Disease Rating Scale, MDS-UPDRS Movement Disorder Society- Unified

UPDRS		MDS-UPDRS	
Tremor scores for each rating scale			
Item – Part II		Item – Part II	
2.16	Tremor	2.10	Tremor
Item – Part III		Item – Part III	
3.20	Rest tremor	3.15	Postural tremor
	Face, RUE, LUE, RLE, LLE		RUE, LUE
3.21	Action tremor	3.16	Kinetic tremor
	RUE, LUE		RUE, LUE
		3.17	Rest tremor
			RUE, LUE, RLE, LLE, lip/jaw
		3.18	Rest constancy
PIGD scores for each rating scale			
Item – Part II		Item – Part II	
2.13	Falling	2.12	Walking and balance
2.14	Freezing	2.13	Freezing
2.14	Walking		
Item – Part III		Item – Part III	
3.29	Gait	3.10	Gait
3.30	Postural stability	3.11	Freezing of gait
		3.12	Postural stability

Parkinson ' s Disease Rating Scale, RUE right upper extremity, LUE left upper extremity, RLE right lower extremity, LLE left lower extremity, PIGD postural instability/gait difficulty.

2. Tremor dominant vs. akinetic-rigid subtypes

The items for tremor dominant *vs.* akinetic-rigid subtypes for corresponding Unified Parkinson's Disease Rating Scale (UPDRS) are shown in Table 2.[1,3] The corresponding items of the MDS-UPDRS were selected to classify the patients accordingly to accommodate the original classification by Eggers et al. Patients were classified as tremor dominant (TD″) if the average tremor score was at least twice the non-tremor score, and vice versa for the akinetic-rigid type. Patients whose denominator was zero but whose mean tremor or non-tremor score was positive were classified as tremor dominant or akinetic-rigid subtype based on numerator values. Patients in whom the ratio scores did not differ by factor two were classified as mixed type.

Table 2. The subscales of UPDRS and MDS-UPDRS for discrimination of tremor dominant vs akinetic-rigid subtypes

UPDRS Unified Parkinson ' s Disease Rating Scale, MDS-UPDRS Movement Disorder Society- Unified

UPDRS		MDS-UPDRS	
Tremor scores for each rating scale			
Item – Part III		Item – Part III	
3.20	Rest tremor	3.15	Postural tremor
	Face, RUE, LUE, RLE, LLE		RUE, LUE
3.21	Action tremor	3.16	Kinetic tremor
	RUE, LUE		RUE, LUE
		3.17	Rest tremor
			RUE, LUE, RLE, LLE, lip/jaw
		3.18	Rest constancy
Non-tremor scores for each rating scale			
Item – Part III		Item – Part III	
3.18	Speech	3.1	Speech
3.19	Facial expression	3.2	Facial expression
3.22	Rigidity	3.3	Rigidity
	Neck, RUE, LUE, RLE, LLE		Neck, RUE, LUE, RLE, LLE
3.27	Arising from chair	3.9	Arising from chair
3.28	Posture	3.13	Posture
3.29	Gait	3.10	Gait
3.30	Postural stability	3.12	Postural stability
3.31	Body bradykinesia and hypokinesia	3.14	Body bradykinesia and hypokinesia

Parkinson ' s Disease Rating Scale, RUE right upper extremity, LUE left upper extremity, RLE right lower extremity, LLE left lower extremity.

References

1. Goetz, C.G., et al. Movement Disorder Society-sponsored revision of the Unified Parkinson's Disease Rating Scale (MDS-UPDRS): scale presentation and clinimetric testing results. *Mov. Disord.* **23**, 2129-2170 (2008)
2. Stebbins, G.T., et al. How to identify tremor dominant and postural instability/gait difficulty groups with the movement disorder society unified Parkinson's disease rating scale: comparison with the unified Parkinson's disease rating scale. *Mov. Disord.* **28**, 668-670 (2013)
3. Eggers, C., et al. Akinetic-rigid and tremor-dominant Parkinson's disease patients show different patterns of FP-CIT single photon emission computed tomography. *Mov. Disord.* **26**, 416-2 (2011)
