# Supplementary material for: A 3-year natural history of orthostatic blood pressure dysregulation in early Parkinson’s disease
Source: NPJ Parkinsons Dis. 2023 Jun 21;9:96. doi: 10.1038/s41531-023-00546-5 (PMC10284855; doi:10.1038/s41531-023-00546-5)
Supplement: Supplementary file 2 — Reporting Summary [file 41531_2023_546_MOESM2_ESM.pdf]

nature portfolio

Corresponding author(s): Joong-Seok Kim

Last updated by author(s): Jun 8, 2023

Reporting Summary

Nature Portfolio wishes to improve the reproducibility of the work that we publish. This form provides structure for consistency and transparency in reporting. For further information on Nature Portfolio policies, see our Editorial Policies and the Editorial Policy Checklist.

Statistics

For all statistical analyses, confirm that the following items are present in the figure legend, table legend, main text, or Methods section.

n/a	Confirmed	
☐	☒	The exact sample size (*n*) for each experimental group/condition, given as a discrete number and unit of measurement
☐	☒	A statement on whether measurements were taken from distinct samples or whether the same sample was measured repeatedly
☐	☒	The statistical test(s) used AND whether they are one- or two-sided<br>*Only common tests should be described solely by name; describe more complex techniques in the Methods section.*
☐	☒	A description of all covariates tested
☐	☒	A description of any assumptions or corrections, such as tests of normality and adjustment for multiple comparisons
☐	☒	A full description of the statistical parameters including central tendency (e.g. means) or other basic estimates (e.g. regression coefficient) AND variation (e.g. standard deviation) or associated estimates of uncertainty (e.g. confidence intervals)
☐	☒	For null hypothesis testing, the test statistic (e.g. F, t, r) with confidence intervals, effect sizes, degrees of freedom and P value noted<br>*Give P values as exact values whenever suitable.*
☒	☐	For Bayesian analysis, information on the choice of priors and Markov chain Monte Carlo settings
☒	☐	For hierarchical and complex designs, identification of the appropriate level for tests and full reporting of outcomes
☒	☐	Estimates of effect sizes (e.g. Cohen's *d*, Pearson's *r*), indicating how they were calculated

Our web collection on statistics for biologists contains articles on many of the points above.

Software and code

Policy information about availability of computer code

Data collection	Data collection was done with Microsoft Excel. The files were then converted into csv files for statistical programs
Data analysis	For data analysis, jamovi software (version 2.3.18) and R software (version 4.2.1) with the PMCMRplus package.

For manuscripts utilizing custom algorithms or software that are central to the research but not yet described in published literature, software must be made available to editors and reviewers. We strongly encourage code deposition in a community repository (e.g. GitHub). See the Nature Portfolio guidelines for submitting code & software for further information.

Data

Policy information about availability of data

All manuscripts must include a data availability statement. This statement should provide the following information, where applicable:

- Accession codes, unique identifiers, or web links for publicly available datasets
- A description of any restrictions on data availability
- For clinical datasets or third party data, please ensure that the statement adheres to our policy

Anonymized data generated during this study are available from the corresponding author on reasonable request from individuals affiliated with research or health care institutions.

Human research participants

Policy information about studies involving human research participants and Sex and Gender in Research.

Reporting on sex and gender	Biological attribute was considered in this study; hence, the term sex was used.
Population characteristics	The mean age at diagnosis was 66.9 ± 9.1 years, and 128 (47.9%) were female. Disease duration at diagnosis was 13.3 ± 10.8 months. The sample is representative of PD population of a single center. It is from our cohort registry that collects data retrospectively and prospectively. Participants with analyzable data were selected from this registry.
Recruitment	PD patients who visited our movement disorder clinics of Seoul St. Mary's Hospital were recruited. As this study enrolled at a single center, this may cause selection bias.
Ethics oversight	This study was approved by the Institutional Review Board of Seoul St. Mary's Hospital.

Note that full information on the approval of the study protocol must also be provided in the manuscript.

Field-specific reporting

Please select the one below that is the best fit for your research. If you are not sure, read the appropriate sections before making your selection.

☒ Life sciences ☐ Behavioural & social sciences ☐ Ecological, evolutionary & environmental sciences

For a reference copy of the document with all sections, see nature.com/documents/nr-reporting-summary-flat.pdf

Life sciences study design

All studies must disclose on these points even when the disclosure is negative.

Sample size	Sample size was not calculated as this study looked into the natural history of enrolled patients. The enrolled PD patients were part of Parkinson's disease cohort of our institution. The authors tried to recruit at least thirty patients for each group to guarantee normality, thus at least 100 PD patients in total.
Data exclusions	Among the subjects, those with missing values were excluded in the relevant statistical tests.
Replication	The results of this study was reproducible. The tests were re-checked with above mentioned statistical software (jamovi, R).
Randomization	As this study is exploratory and observational study, randomization was not performed. Covariate was not adjusted as the aim of the study was to look into the natural course of the disease subtypes.
Blinding	Blinding was not relevant in this study because of its design (an observatory cohort).

Reporting for specific materials, systems and methods

We require information from authors about some types of materials, experimental systems and methods used in many studies. Here, indicate whether each material, system or method listed is relevant to your study. If you are not sure if a list item applies to your research, read the appropriate section before selecting a response.

Materials & experimental systems

n/a	Involved in the study	
☒	☐	Antibodies
☒	☐	Eukaryotic cell lines
☒	☐	Palaeontology and archaeology
☒	☐	Animals and other organisms
☐	☒	Clinical data
☒	☐	Dual use research of concern

Methods

n/a	Involved in the study	
☒	☐	ChIP-seq
☒	☐	Flow cytometry
☒	☐	MRI-based neuroimaging

Clinical data

Policy information about clinical studies

All manuscripts should comply with the ICMJE guidelines for publication of clinical research and a completed CONSORT checklist must be included with all submissions.

Clinical trial registration	*Provide the trial registration number from ClinicalTrials.gov or an equivalent agency.*
Study protocol	Study protocol is not available as this study is founded upon a PD registry cohort.
Data collection	Two hundred sixty-seven drug-naïve and de novo PD patients presenting between August 2012 and July 2020 were enrolled in this cohort. Data was collected from enrolled patients who visited the St.Mary's Hospital.
Outcomes	The outcome was how the subtypes of Parkinson's disease changed with progression. The subtypes of PD was defined by the results of head-up tilt tests. As the main focus was the natural history, descriptive statics were applied to observe the flow of changes. In cases of group comparisons, analysis of variance (or Kruskal-Wallis test was performed for continuous or ordinal variables when appropriate. Categorical variables were examined by Fisher's exact test. Cochran-Armitage test and Jonckheere-Terpstra test were executed.
